# Outcomes of patients lost to follow‐up after antiretroviral therapy initiation in rural north‐eastern South Africa

**DOI:** 10.1111/tmi.13236

**Published:** 2019-04-09

**Authors:** Julie Ambia, Chodziwadziwa Kabudula, Kathryn Risher, Francesc Xavier Gómez‐Olivé, Brian D. Rice, David Etoori, Georges Reniers

**Affiliations:** ^1^ Department of Population Health London School of Hygiene & Tropical Medicine London UK; ^2^ MRC/Wits Rural Public Health and Health Transitions Research Unit (Agincourt) School of Public Health University of the Witwatersrand Johannesburg South Africa; ^3^ MeSH Consortium Department of Public Health Environments and Society Faculty of Public Health and Policy London School of Hygiene and Tropical Medicine London UK

**Keywords:** HIV/AIDs, loss to follow‐up, re‐engagement, competing risk analysis, record linkage, demographic surveillance system, VIH/SIDA, perte au suivi, réengagement, analyse des risques concurrents, couplage des données, système de surveillance démographique

## Abstract

**Objective:**

The vital status of patients lost to follow‐up often remains unknown in antiretroviral therapy (ART) programmes in sub‐Saharan Africa because medical records are no longer updated once the patient disengages from care. Thus, we aimed to assess the outcomes of patients lost to follow‐up after ART initiation in north‐eastern South Africa.

**Methods:**

Using data from a rural area in north‐eastern South Africa, we estimated the cumulative incidence of patient outcomes (i) after treatment initiation using clinical records, and (ii) after loss to follow‐up (LTFU) using data from clients that have been individually linked to Agincourt Health and Demographic Surveillance System (AHDSS) database. Aside from LTFU, we considered mortality, re‐engagement and migration out of the study site. Cox proportional hazards regression was used to identify covariates of these patient outcomes.

**Results:**

Between April 2014 and July 2017, 3700 patients initiated ART and contributed a total of 6818 person‐years of follow‐up time. Three years after ART initiation, clinical record‐based estimates of LTFU, mortality and documented transfers were 41.0% (95% CI: 38.5–43.4%), 1.9% (95% CI 1.0–3.2%) and 0.1% (95% CI 0.0–0.9%), respectively. Among those who were LTFU, the cumulative incidence of re‐engagement, out‐migration and mortality at 3 years were 38.1% (95% CI 33.1–43.0%), 49.4% (95% CI 43.1–55.3%) and 4.7% (95% CI 3.5–6.2%), respectively. Pregnant or breastfeeding women, foreigners and those who initiated ART most recently were at an increased risk of LTFU.

**Conclusion:**

LTFU among patients starting ART in north‐eastern South Africa is relatively high and has increased in recent years as more asymptomatic patients have initiated treatment. Even though this tendency is of concern in light of the prevention of onwards transmission, we also found that re‐engagement in care is common and mortality among persons LTFU relatively low.

## Abstract (fr)

**Objectif:**

Le statut vital des patients perdus au suivi reste souvent inconnu dans les programmes de traitement antirétroviral (ART) en Afrique subsaharienne parce que les dossiers médicaux ne sont plus mis à jour une fois que le patient se désengage des soins. Notre objectif était d’évaluer les résultats des patients dans le nord‐est de l'Afrique du Sud.

**Méthodes:**

A l'aide de données provenant d'une zone rurale du nord‐est de l'Afrique du Sud, nous avons estimé l'incidence cumulée des résultats pour les patients (i) après le début du traitement à l'aide des dossiers cliniques et (ii) après la perte au suivi (PS) à l'aide des données des patients qui ont été reliées individuellement à la base de données du système de surveillance démographique et de santé (AHDSS) d'Agincourt. Outre les PS, nous avons pris en compte la mortalité, le réengagement et la migration hors du site de l’étude. La régression des risques proportionnels de Cox a été utilisée pour identifier les covariables de ces résultats pour le patient.

**Résultats:**

Entre avril 2014 et juillet 2017, 3.700 patients ont commencé l’ART constituant un suivi total de 6.818 années‐personnes. Trois ans après le début de l’ART, les estimations des PS, de la mortalité et des transferts documentés selon les registres cliniques étaient de 41,0% (IC95%: 38,5% à 43,4%), 1,9% (IC95%: 1,0% à 3,2%) et 0,1% (IC95%: 0,0% ‐0,9%), respectivement. Parmi ceux qui étaient PS, l'incidence cumulative de réengagement, d’émigration et de mortalité à trois ans était de 38,1% (IC95%: 33,1% à 43,0%), 49,4% (IC95%: 43,1% à 55,3%) et 4,7% (IC95%: 3,5% ‐6,2%), respectivement. Les femmes enceintes ou allaitantes, les étrangers et les personnes qui ont commencé l’ART le plus récemment couraient un risque accru de PS.

**Conclusion:**

La PS chez les patients commençant une ART dans le nord‐est de l'Afrique du Sud est relativement élevée et a augmenté ces dernières années à mesure que davantage de patients asymptomatiques ont commencé le traitement. Même si cette tendance est préoccupante à la lumière de la prévention de la transmission, nous avons également constaté que le réengagement dans les soins était courant et que la mortalité parmi les PS était relativement faible.

## Introduction

Sustained use of antiretroviral therapy (ART) among HIV‐infected patients reduces HIV viral load to undetectable levels, slows the progression of HIV and reduces the risk of onwards sexual transmission 1, 2. However, high rates of loss to follow‐up (LTFU) have been reported in many HIV care and treatment programmes in sub‐Saharan Africa 3, 4, 5. Perception of good health, use of alternative medicine, stigma, treatment fatigue, lack of knowledge, transport fare and competing demands for time are some of the factors that are known to contribute to disengagement 6. However, not all patients deemed LTFU have stopped taking ART, as some patients switch facilities 7, 8. Thus it is important to better understand the vital and treatment status of the LTFU patients both for improving the delivery of HIV services and for estimating the impact of ART on HIV‐associated mortality.

Several strategies have been employed to obtain information on the vital status of patients LTFU, including patient tracing, the review of obituaries in newspapers and record linkage with Civil Registration and Vital Statistics 9, 10. The IeDEA Network used sample‐based tracing to locate LTFU by phone or home visits 8, 11, 12.

In this study, we used information from clinic records that are individually linked with data from the Agincourt Health and Demographic Surveillance System (AHDSS) 13 in South Africa to ascertain both vital and migration status of patients who are LTFU. The link with the AHDSS also allowed us to identify patients who are LTFU at one facility and subsequently registered at another facility that serves the AHDSS population. We use these data to estimate outcomes of patients after (i) treatment initiation and (ii) LTFU.

## Methods

### Ethics statement

The study was approved by the University of the Witwatersrand Human Research Ethics Committee and the London School of Hygiene and Tropical Medicine Research Ethics Committee. Data analysis was conducted using anonymised data.

### Study setting and population

The AHDSS study area covers 475 km^2^ in Bushbuckridge, Mpumalanga province, north‐eastern South Africa. The AHDSS has been tracking demographic and health events in people living in the HDSS boundary since 1992. As of 2014, the population was approximately 115 000 individuals living in 17 000 households spread across 31 villages 14. Overall, 47.9% of the population are men and 52.1% are women 15. One third of the population are former Mozambican refugees who moved into the area during the civil war that began in 1977 and ended in 1992. About 9 in 10 of the former Mozambican refugees have now attained South African citizenship 16.

A cross‐sectional biomarker survey conducted in 2010–2011 estimated that one in five adults in the AHDSS population were HIV‐infected 17. The likelihood of being diagnosed with HIV was higher among South Africans than foreigners 17. HIV prevalence was higher among women (23.9%) than men (10.6%), consistent with other countries in sub‐Sahara Africa (SSA) 18. However, temporary labour migration is quite high among young adult men in the study site 19 and they may be under‐represented in the AHDSS serosurvery 20.

**Figure 1:**
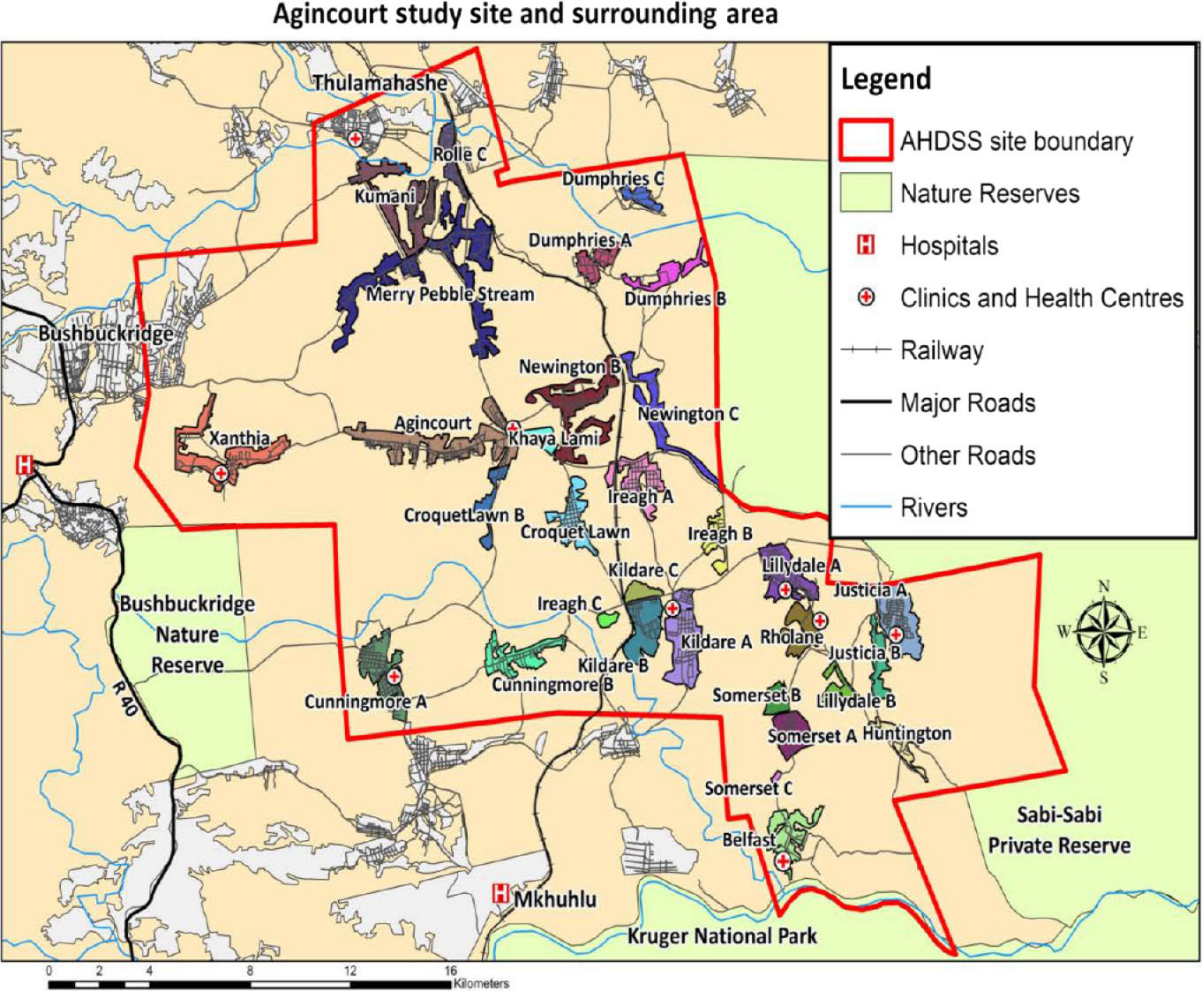
*Map of agincourt health and demographic surveillance system*. [Colour figure can be viewed at wileyonlinelibrary.com]

ART provision began in 2004 in two secondary hospitals, between 25 and 60 km away from the AHDSS study area 13. A community health centre, Bhubezi, was opened in 2007 in the AHDSS study area and was the first to provide HIV care and treatment locally. Currently, HIV care and treatment services have been decentralised to 10 primary health care facilities in the AHDSS study area (Figure 1). Initiation of ART among persons newly diagnosed with HIV at these facilities is in line with national antiretroviral therapy guidelines. Since December 2014, these recommended ART initiation to all pregnant or breastfeeding women and any patient with a CD4 count <500/μL 21. In September 2016, the treatment eligibility criteria were further revised to include all persons diagnosed with HIV 22.

To promote adherence two non‐governmental organisations conduct tracing of patients who disengage from care (Right to Care and Home‐Based Carers (HBCs)) in the AHDSS catchment area. To that end, nurses at the clinic prepare a list of patients LTFU and a Right to Care linkage officer contacts these patients by phone. If a patient cannot be traced by phone, the case is referred to HBCs for physical tracing.

### Data

The core data infrastructure for this study consists of HIV patient records that are linked to the AHDSS using Point‐of‐Contact Interactive Record Linkage (PIRL), a procedure that has been described elsewhere 23. In brief, a field worker is stationed at the clinic reception area to inform all new patients about the study objectives and record linkage procedures. Patients who consent are asked to declare a number of personal identifiers that are used to search a local copy of the AHDSS database using a probabilistic algorithm. Matches are confirmed in interaction with the patients, and the names of other household members are used as a key attribute to adjudicate between possible matches. The PIRL software tool also contains a provision to log follow‐up visits 24.

Record linkage started in April 2014, at seven government facilities and in 2016 this was extended to include one additional health facility. In this study, we only included patients who had been linked before or at the time of ART initiation. From these eight health facilities, we extracted the following information from the clinic records; clinic visit dates, date of HIV diagnosis, date of ART initiation, baseline CD4 counts, WHO clinical staging, reason for ART initiation, pregnancy status, vital status (i.e. alive or dead from any cause), date of death and availability of a duly completed facility transfer form. The AHDSS database contains information obtained from an annual household visits 13, including changes in residency and the vital status of all household members.

### Operational definitions

A patient who had commenced ART was considered LTFU if they failed to return within 90 days of a scheduled appointment. Our analyses were conducted in two stages. The first set of analyses only make use of clinic attendance records, to prospectively follow patients from the date of ART initiation to either LTFU, a documented transfer, death or administrative censoring on the date of data extraction from clinical records (i.e. 15 July 2017). The second set of analyses make use of linked AHDSS and clinic attendance records to evaluate the outcomes of the patient who were deemed LTFU on the basis of their clinical record. In this second set of analyses, time was measured from the date of last clinic visit to the earliest of the following events: re‐engagement, out‐migration from the AHDSS, death or administrative censoring on the date of last AHDSS household visit.

An individual was considered to have migrated if he or she was reported in the AHDSS round to have permanently left the surveillance area. The date of migration is reported retrospectively by relatives and there may be some misreporting. Thus, all those who reported to have migrated just before their last clinic visit are here treated as if they migrated on the day of their last clinic visit. Re‐engagement was defined as the resumption of clinic visits in the same clinic or another clinic within the AHDSS surveillance area. The latter is sometimes also referred to as ‘silent transfers’ 12. The identification of silent transfers is facilitated through record linkage to the AHDSS. National origin was based on the country of father's birth (i.e. South African or foreigner). Late presentation was a composite of CD4 counts of <250 cells/μL and WHO stage III or IV at time of ART initiation.

### Data analysis

In our first set of analyses, we used competing risks survival analysis to estimate the cumulative incidence of (i) LTFU, (ii) all‐cause mortality (as reported in clinic records only) and (iii) documented transfers following treatment initiation. The complement of these three probabilities represents the proportion of patients who were alive and still on ART in the same facility where they initiated treatment without interruptions in excess of 90 days following the last scheduled visit. For this analysis, we only use data from the clinic visit logs. To impute a return date, we added 30 days to all patients who never returned after initiating ART (as their clinic return dates were missing).

Our second set of analyses focuses on patients deemed LTFU, and relies on data from both clinic visit logs and the AHDSS. Again, we conduct competing risks survival analysis to estimate the cumulative incidence of (i) re‐engagement, (ii) all‐cause mortality and (iii) out‐migration at or after LTFU. The complement of these three probabilities represents the proportion of patients that were LTFU but still alive, living in the study site and not known to be on treatment at another facility.

Covariates of the outcomes of interest are assessed using semi‐parametric Cox regression models. Again, we first model LTFU after treatment initiation. Patients’ exposure time is ended at the time that they become LTFU, die or formally transfer out and right censored at the date of the clinic data extraction (15 July 2017). A similar approach is used for modelling the covariates of patient outcomes following LTFU. As stipulated above, we consider re‐engagement with HIV care, out‐migration from the study area and death as competing risks. Covariates with *P*‐value below 0.05 in bivariate analyses were included in the adjusted model. Schoenfeld residuals were used to evaluate the proportional hazards assumption (Figure S1–S4). Wherever the proportional hazards assumption was violated, we included a linear interaction between the covariate and analysis time. Data were analysed using Stata 14.0 (College Station, Texas, USA).

## Results

Table 1 illustrates the characteristics of 3700 patients who were linked to an AHDSS record and initiated ART between 30 April 2014 and 15 July 2017. Majority of the patients (88.8%; 3700/4168) who reported residency in one of the AHDSS villages could be linked to an AHDSS record. The attributes of linked and unlinked records are further explored in a table in the supporting information (Table S1). All of these patients were reportedly ART naïve, but we cannot exclude that some previously received treatment elsewhere without declaring it as such. At the time of ART initiation less than a quarter of the patients were men and one‐fifth of the patients were pregnant or breastfeeding. The median age at ART initiation was 38.7 years [Interquartile range (IQR): 31.9–46.0 years] among men, 34.3 years [IQR: 27.1–44.9 years] among non‐pregnant women and 27.1 years [IQR: 23.4–31.6 years] among pregnant or breastfeeding women. Overall, 47.1% (1733/3681) of individuals presented late for care. The highest percentage of late presentation was observed among men (67%; 576/866), followed by non‐pregnant women (46%; 950/2077), and pregnant or breastfeeding women (28%; 207/738). The 3700 patients jointly contributed a total of 6818 person‐years of observation time to the analyses. Of the 966 patients LTFU, 3.8% were lost within 1 year of ART initiation, 25.9% within 2 years and 81.9% within 3 years.

**Table 1:** Characteristics of linked patients initiating ART between April 2014 and July 2017 in the Agincourt sub‐district in Mpumalanga Province, South Africa

Patient characteristic	*n*	%
Sex & pregnancy status		
Men	874	23.6
Women (non‐pregnant)	2084	56.3
Women (pregnant or breastfeeding)	742	20.1
Missing	0	
National origin		
South African	2457	66.5
Foreigner	1240	33.5
Missing	3	
Age at ART initiation (in years)		
<20	220	6.0
20–29	1177	31.9
30–39	1160	31.4
40–49	631	17.0
≥50	507	13.7
Missing	5	
Year of ART initiation		
2014	634	17.1
2015	1223	33.0
2016	1282	34.7
2017	561	15.2
Missing	0	
Late ART initiation		
No	1948	52.9
Yes	1733	47.1
Missing	19	
Number of patients contributing to		
<1 year of exposure time to the analysis	629	17.0
1–2 years of exposure time to the analysis	1225	33.1
2–3 years of exposure time to the analysis	1285	34.7
>3 years of exposure time to the analysis	561	15.2
Missing	0	
Number of patients LTFU since ART initiation		
<1 years	37	3.8
1–2 years	213	22.0
2–3 years	541	56.0
>3 years	175	18.1
Missing	0	
Total	3700	100

### Patient status after ART initiation

**Figure 2:**
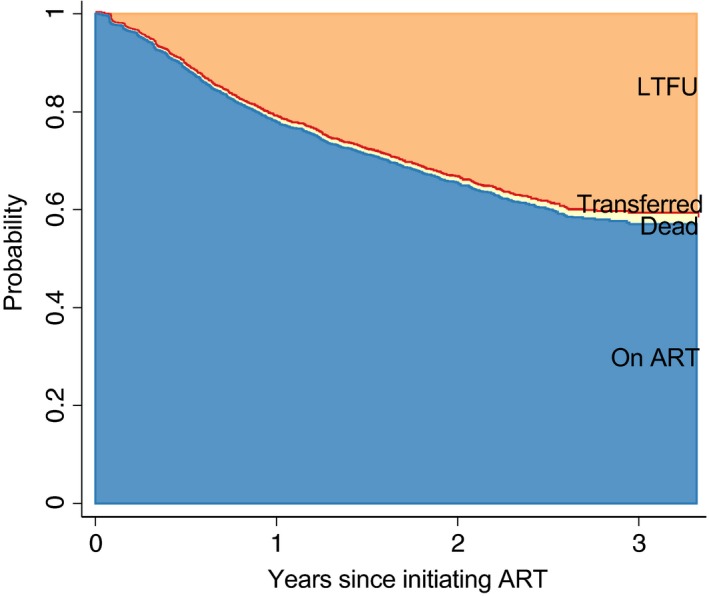
*Patient outcomes following ART initiation using clinic attendance records, Agincourt (2014–2017)*. [Colour figure can be viewed at wileyonlinelibrary.com]

Figure 2 and Table S2 show the probabilities of patients being retained in care (*n* = 2704), dying (*n* = 28), LTFU (*n* = 966) or transferring (*n* = 4). Using exclusively clinic visit attendance records, the cumulative incidence of LTFU, mortality and documented transfers at 1 year after initiating ART were estimated at, 21.3% (95% CI 19.9–22.8%), 0.67% (95% CI 0.43–1.02%) and 0.13% (95% CI 0.04–0.86%), respectively. After 3 years, 41.0% (95% CI 38.5–43.4%) were LTFU, 1.88% (95% CI 1.03–3.17%) had died and 0.13% (95% CI 0.04–0.86%) were known to have transferred to another facility.

### Patient status after LTFU

**Figure 3:**
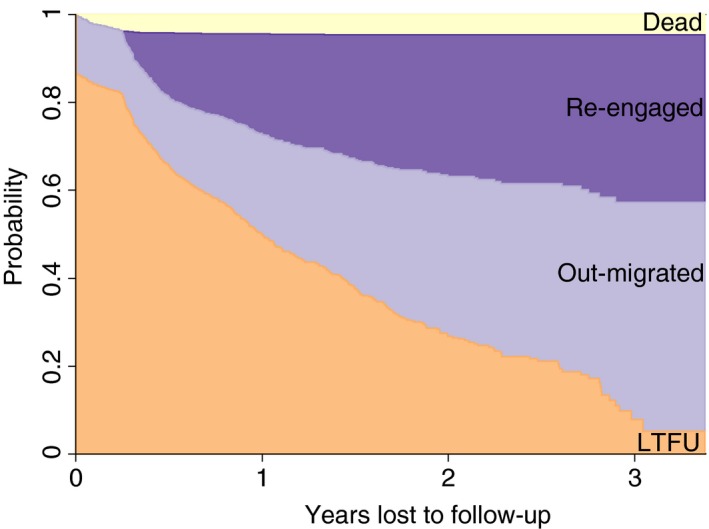
*Patient outcomes after LTFU ascertained through record linkage between clinic records and AHDSS, Agincourt (2014–2017)*. [Colour figure can be viewed at wileyonlinelibrary.com]

Figure 3 and Table S3 summarise the cumulative incidence of re‐engagement (*n* = 267), out‐migration (*n* = 298) and mortality (*n* = 44) after LTFU based on the linked clinic‐AHDSS database. The cumulative incidence of re‐engagement increased from 23.0% (95% CI 20.3–25.8%) at 1 year to 38.1% (95% CI 33.1–43.0%) at year 3. The cumulative incidence of out‐migration increased from 22.8% (95% CI 20.1–25.5%) in year 1 to 49.4% (95% CI 43.1–55.3%) in year 3. About 15% who out‐migrated at time 0 are those who had migrated between the date of last clinic visit and the date at which they are considered LTFU. The cumulative incidence of mortality increased marginally from 4.5% (95% CI 3.3–6.0%) at year 1 to 4.7% (95% CI 3.5–6.2%) at year 3. The treatment status of the remaining 37% (357/966) could not be ascertained using the linked clinic‐AHDSS dataset. After 3 years, about 10% of the patients were LTFU. These are patients who interrupted care at the place where they first initiated ART, are still living in the study area, and are not known to re‐engaged at the same facility or transferred to another facility. The current treatment and vital status of patients who migrated out of the AHDSS area is also unknown.

Of the patients who resumed HIV care, 43.8% re‐engaged in the same health facility, 53.9% continued treatment at another health facility and 2.2% continued care at two facilities in the AHDSS area. The cumulative incidence of transferring to another facility in the study site was 3.2% (95% CI 2.2–4.4%) at 1 year and 14.4% (95% CI 10.8–18.6%) at year 3.

### Predictors of LTFU after ART initiation

Table 2 summarises the output of the regression model of LTFU following ART initiation. In both crude and adjusted analysis, a linear interaction with time was added to the model whenever the proportional hazards assumption was violated. In many of these cases, the interaction term suggests that initial differences between patient subgroups decline as time goes on. Results of the tests for the proportional hazards assumption are presented in Table S4 and Figure S1 in the supporting information.

**Table 2:** Factors associated with LTFU after ART initiation

	LTFU	
	Crude HR (95% CI)	Adjusted HR^a^ (95% CI)
Sex & pregnancy status		
Women (non‐pregnant)	1	1
Pregnant or breastfeeding women	**2.64 (2.07–3.38)**	**2.53 (1.95–3.27)**
Time‐varying	**0.69 (0.53–0.89)**	**0.61 (0.46–0.79)**
Men	1.17 (0.89**–**1.52)	1.21 (0.92**–**1.58)
Time‐varying	**1.28 (1.01–1.63)**	**1.27 (1.00–1.63)**
National origin		
South African	1	1
Foreigner	**1.39 (1.11–1.73)**	**1.41 (1.13–1.75)**
Time‐varying	**0.74 (0.59–0.92)**	**0.67 (0.53–0.83)**
Age at ART initiation		
<20 years	**2.16 (1.29–3.60)**	1.61 (0.95**–**2.72)
Time‐varying	**2.02 (1.30–3.14)**	**2.44 (1.56–3.84)**
20–29 years	**2.63 (1.72–4.01)**	**1.89 (1.22–2.94)**
Time‐varying	0.92 (0.61**–**1.37)	1.20 (0.79**–**1.82)
30–39 years	**2.04 (1.32–3.16)**	**1.70 (1.09–2.64)**
Time‐varying	0.77 (0.50**–**1.17)	0.86 (0.57**–**1.31)
40–49 years	**1.92 (1.19–3.09**)	**1.76 (1.09–2.83)**
Time‐varying	0.84 (0.53**–**1.34)	0.88 (0.55**–**1.39)
≥50 years	1	1
Year of ART initiation		
2014	1	1
2015	**1.62 (1.17–2.23)**	**1.71 (1.24–2.36)**
Time‐varying	**0.65 (0.49–0.86)**	**0.68 (0.51–0.90)**
2016	**3.17 (2.15–4.66)**	**3.41 (2.31–5.02)**
Time‐varying	**0.19 (0.11–0.34)**	**0.21 (0.12–0.36)**
2017	**4.74 (1.58–14.27)**	**5.16 (1.71–15.53)**
Time‐varying	**0.00 (0.00–0.02)**	**0.00 (0.00–0.02)**
Late ART initiation		
No	1	
Yes	0.90 (0.80**–**1.03)	

a LTFU: 
No of subjects – 3692 No of failures – 964 Time at risk – 4621

Figures in bold showed statistical associations.

Pregnant or breastfeeding women had a more than twofold increased risk of LTFU (adjusted hazard ratio (AHR): 2.53; 95% CI 1.95–3.27) compared with non‐pregnant women, but their elevated risk of LTFU attenuated by 39% every year. Men's risk of LTFU was initially not different from that of non‐pregnant women, but increased by 27% each calendar year. Former Mozambican settlers had a 41% higher risk of LTFU (AHR: 1.41; 95% CI 1.13–1.75) at time zero and it declined by 33% every year.

LTFU risks for persons under 50 years of age were initially comparable, but disparities increased over time with younger men and women experiencing higher LTFU risks at longer durations. Men and women who initiated ART in later calendar years (i.e. 2015, 2016 and 2017) were at a higher risk of LTFU, but the interactions with time all suggest that the differences decline as the time since starting ART increases.

### Predictors of re‐engagement, mortality and out‐migration after LTFU

Table 3 summarises crude and adjusted hazard estimates of re‐engagement, mortality and out‐migration following LTFU from Cox regression models. The proportional hazards assumption was violated in two instances, and this was accommodated by the inclusion of a linear interaction between the covariate of interest and analysis time (Table S5 and Figures S2–S4). The interaction between age and analysis time on mortality is only included in the supporting information (Table S6) as it led to very large coefficient estimates due to quasi‐separation in the data.

**Table 3:** Factors associated with re‐engagement, mortality and out‐migration following LTFU

	Re‐engagement		Mortality		Out‐migration	
	Crude HR (95% CI)	Adjusted HR^a^ (95% CI)	Crude HR (95% CI)	Adjusted HR^b^ (95% CI)	Crude HR (95% CI)	Adjusted HR^c^ (95% CI)
Sex & pregnancy status						
Women (non‐pregnant)	1	1	1	1	1	1
Pregnant or breastfeeding women	**0.58 (0.43–0.79)**	**0.61 (0.44–0.84)**	**0.27 (0.09–0.79)**	0.69 (0.22–2.11)	1.03 (0.80–1.33)	1.02 (0.79–1.32)
Men	1.02 (0.77–1.35)	1.03 (0.77–1.39)	1.36 (0.73–2.53)	0.85 (0.45–1.60)	**0.65 (0.47–0.89)**	**0.65 (0.48–0.90)**
National origin						
South African	1	1	1		1	
Foreigner	**0.69 (0.53–0.90)**	**0.68 (0.51–0.89)**	1.32 (0.72–2.40)		1.11 (0.88–1.40)	
Age at ART initiation						
<20 years	**0.49 (0.28–0.86)**	0.58 (0.32–1.04)	**0.08 (0.02–0.33)**	**0.12 (0.03–0.54)**	1.64 (0.86–3.13)	
20–29	0.64 (0.40–1.01)	0.77 (0.47–1.25)	**0.05 (0.02–0.16)**	**0.08 (0.03–0.26)**	1.79 (0.99–3.23)	
30–39	0.69 (0.43–1.11)	0.77 (0.47–1.25)	**0.26 (0.12–0.55)**	**0.28 (0.13–0.60)**	1.57 (0.86–2.89)	
40–49	0.85 (0.51–1.41)	0.84 (0.50–1.41)	**0.33 (0.14–0.73)**	**0.33 (0.15–0.74)**	1.24 (0.64–2.41)	
≥50 years	1	1	1	1	1	
Year of ART initiation						
2014	1	1	1		1	1
2015	**0.60 (0.37–0.96)**	**0.56 (0.34–0.90)**	0.85 (0.44**–**1.65)		1.09 (0.82–1.46)	1.08 (0.81–1.44)
Time‐varying	1.75 (0.98–3.14)	**1.81 (1.01–3.26)**				
2016	**0.22 (0.12–0.42)**	**0.20 (0.10–0.39)**	0.48 (0.20**–**1.14)		**1.51 (1.09–2.07)**	**1.48 (1.07–2.04)**
Time‐varying	**7.41 (3.11–17.71)**	**8.47 (3.52–20.36)**				
Late ART initiation						
No	1	1	1	1	1	
Yes	**1.28 (1.01**–**1.63)**	1.02 (0.79–1.33)	**7.86 (3.32**–**18.60)**	**5.37 (2.21**–**13.04)**	0.91 (0.72–1.15)	

a Re‐engagement: 
No of subjects – 960 No of failures – 265 Time at risk – 852

b Mortality: 
No of subjects – 960 No of failures – 44 Time at risk – 852

c Out‐migration: 
No of subjects – 966 No of failures – 298 Time at risk – 856

Figures in bold showed statistical associations.

Initiating ART during pregnancy/breastfeeding (AHR: 0.61; 95% CI 0.44–0.84) or being a foreigner (AHR 0.68; 95% CI 0.51–0.89) were associated with a reduction in the risk of returning to care. Men and women who initiated ART in later calendar years (i.e. 2015 and 2016) were at a lower risk of returning to care, and the low risk intensified in the most recent period.

In univariate analysis, pregnant or breastfeeding women had a 73% lower hazard (HR 0.27; 95% CI 0.09–0.79) of dying than non‐pregnant women. After adjustment of other covariates, the relationship was no longer statistically significant. After adjustment, older age (≥50 years) was associated with an increase in mortality risk. The relative hazard of dying was higher among patients who presented late for ART initiation (AHR 5.34; 95% CI 2.20–12.96) than in patients who presented early (WHO clinical stage I or II or with CD4 counts of >251 cells/μL).

In multivariate analysis, men (AHR: 0.65; 95% CI 0.48–0.90) were at a lower risk of migrating out of AHDSS than women. Patients who initiated ART in later years (i.e. 2015 and 2016) were also more likely to migrate out of the study area than those who initiated in 2014.

## Discussion

Using data from eight clinics in north‐eastern South Africa (Agincourt), we estimated that 21% and 41% of the patients were LTFU at 12 and 36 months after treatment initiation, respectively. The percentage of deaths and transfers to other facilities within 3 years that were recorded in the medical records were 2% and 0.1%, respectively. Our estimates of LTFU are higher than those in studies from Johannesburg (14% at 12 months) 25 and Tanzania (18% at 12 months) 26.

It is possible that LTFU rates have increased as a result of ART programmes expanding to include all persons newly diagnosed with HIV regardless of their CD4 cell count or clinical stage, thereby including a larger number of patients who initiate ART when they are still asymptomatic 27. This also offers an explanation for the higher LFTU rates among patients who initiated treatment in more recent calendar years.

Our finding that men and pregnant or breastfeeding women were at an increased risk of LTFU corroborates the results from other studies in sub‐Saharan Africa 28, 29, 30, 31. For pregnant women in particular, it has been hypothesised that they are more likely to disengage from care because they are often asymptomatic and discontinue treatment once the risk of onwards transmission to her child has dissipated 32. Women may also be more mobile to seek the support of her relatives around or after childbirth and that may also affect her health seeking behaviour.

Using linked clinic‐HDSS data, we were in position to evaluate the outcomes of patients after they disengaged from ART services. Of the patients who were LTFU, 38% had re‐engaged within 3 years, 49% migrated out of the HDSS area and 5% had died. Consistent with two systematic reviews of tracing studies 7, 33, death was the least important cause of LTFU in this cohort. It is also worth noting that mortality within 24 months of LTFU is lower than that reported in tracing studies in the Zambia where 8% had died within 2 years 34, and in East Africa where 13% had died within 3 years 8. There are two possible explanations for these differences. On the one hand, these discrepancies with earlier tracing studies could be explained by declining mortality among patients LTFU over time. Even though our results do indeed point in that direction, they did not reach statistical significance by conventional standards. They are, however, plausible and could be attributed to a combination of factors including the availability of more effective ART regimens that are more forgiving of lapses in treatment and changes in ART initiation threshold guidelines, which mean patients increasingly initiate ART when they are still asymptomatic 22, 35, 36. However, we cannot exclude that the disparities with earlier tracing studies are due to the fact that we could not establish the vital status of patient who had migrated out of the HDSS area. Even though migration is generally correlated with good health 37, this could be a source of bias in our estimates. Interestingly, men were less likely to have migrated than women, but we have no data to explore this association further.

We found that 14% of the patients had transferred to another facility at 36 months. Similar levels of transfers have been reported other African studies. In an individual patient data meta‐analysis of nine tracing studies conducted in SSA, 15% of the patients had transferred to another facility at the third year 38. A previous study cited the decentralisation of treatment and the proximity to new health facility offering ART as the main reason for transferring 11.

### Study limitations

This study was done in one rural community in north‐east South Africa, thus it may not be possible to generalise these findings. Second, ongoing studies such as the Nkateko health service trial in this area may have affected the study results. In brief, the main objective of this trial was to enhance adherence to clinic visits using phone‐based reminders and home visits by lay health workers among patients with hypertension 39. Of note, some of the hypertensive patients were also HIV‐infected and thus benefited by being continually reminded of their clinic visits. Third, clinical data that was used in this study was obtained from patient attendance records and this restricted the variables that were available for analysis. Fourth, some patients may have been enrolled in differentiated care models that did not require them to visit clinics with the same frequency. Failing to account for these patients distinct visit patterns may have resulted to an overestimation of LTFU in this study. However, the rollout of differentiated care only started in late 2016, thus the number of patients enrolled in differentiated care model may have been negligible. Last, we did not quantify the quality of data linkage. A study in Tanzania that used a similar data linkage approach, managed to link 84% of the patients who declared residency in the HDSS area to an HDSS record 23.

## Conclusion

As observed in clinical cohort studies in other African populations, LTFU among patients starting ART in north‐eastern South Africa is relatively high. LTFU rates are typically higher among pregnant women but are also increasing in other patient subgroups as treatment eligibility criteria have expanded and patients increasingly start ART shortly after diagnosis when they are still asymptomatic. Whereas this tendency is of concern in light of the prevention of onwards transmission, we also found that re‐engagement in care is common (40% within 3 years) and mortality relatively low (5% within 3 years). The LTFU and patient mobility rates identified in this population further suggest that surveillance methods that solely rely on clinic records without unique patient identifiers will be severely limited to accurately represent treatment coverage.

## Supporting information


**Figure S1.** Predicted hazards of LTFU and 95% CI.
**Figure S2.** Predicted hazards of re‐engagement and 95% CI.
**Figure S3.** Predicted hazards of mortality and 95% CI.
**Figure S4.** Predicted hazards of out‐migration and 95% CI.
**Table S1.** Characteristics of linked and unlinked patients initiating ART between April 2014 and July 2017 in the Agincourt sub‐district in Mpumalanga Province, South Africa
**Table S2.** Patient outcomes following ART initiation using clinic attendance records
**Table S3.** Patient outcomes after LTFU ascertained through record linkage with the AHDSS
**Table S4.** Patient outcomes following ART initiation proportional hazard assumption test
**Table S5.** Patient outcomes after LTFU proportional hazard assumption test
**Table S6.** Interaction between age and analysis time on mortality
