# Supplementary material for: Outcomes of patients lost to follow‐up after antiretroviral therapy initiation in rural north‐eastern South Africa
Source: Trop Med Int Health. 2019 Apr 9;24(6):747–56. doi: 10.1111/tmi.13236 (PMC6563456; doi:10.1111/tmi.13236)
Supplement: Supplementary file 1 — Figure S1. Predicted hazards of LTFU and 95% CI. Figure S2. Predicted hazards of re‐engagement and 95% CI. Figure S3. Predicted hazards of mortality and 95% CI. Figure S4. Predicted hazards of out‐migration and 95% CI. Table S1. Characteristics of linked and unlinked patients initiating ART between April 2014 and July 2017 in the Agincourt sub‐district in Mpumalanga Province, South Africa Table S2. Patient outcomes following ART initiation using clinic attendance records Table S3. Patient outcomes after LTFU ascertained through record linkage with the AHDSS Table S4. Patient outcomes following ART initiation proportional hazard assumption test Table S5. Patient outcomes after LTFU proportional hazard assumption test Table S6. Interaction between age and analysis time on mortality [file TMI-24-747-s001.docx]

**Outcomes of patients lost to follow-up after antiretroviral therapy initiation in rural north-eastern South Africa**

**Supplementary Digital Content 1**

Table S1. Characteristics of linked and unlinked patients initiating ART between April 2014 and July 2017 in the Agincourt sub-district in Mpumalanga Province, South Africa

| **Characteristic** | **Total** | **Linked (%)** | **Unlinked (%)** | **P values** |
| --- | --- | --- | --- | --- |
| **Sex & pregnancy status** |  |  |  | 0.040 |
| Men | 1,009 | 86.6 | 13.4 |  |
| Women (non-pregnant) | 2,334 | 89.3 | 10.7 |  |
| Women (pregnant or breastfeeding) | 825 | 89.9 | 10.1 |  |
| Missing | 0 |  |  |  |
| **Marital status** |  |  |  | 0.031 |
| Married | 1,237 | 87.1 | 12.9 |  |
| Single | 2,310 | 90.3 | 9.7 |  |
| Widowed | 182 | 90.1 | 9.9 |  |
| Divorced | 58 | 91.4 | 8.6 |  |
| Missing | 378 |  |  |  |
| **Age at ART initiation (in years)** |  |  |  | 0.731 |
| <20 | 253 | 87.0 | 13.0 |  |
| 20-29 | 1,334 | 88.2 | 11.8 |  |
| 30-39 | 1,305 | 88.9 | 11.1 |  |
| 40-49 | 706 | 89.4 | 10.6 |  |
| ≥50 | 565 | 89.7 | 10.3 |  |
| Missing | 5 |  |  |  |
| **Year of ART initiation** |  |  |  | <0.001 |
| 2014 | 691 | 91.8 | 8.2 |  |
| 2015 | 1,370 | 89.3 | 10.7 |  |
| 2016 | 1,425 | 90.0 | 10.0 |  |
| 2017 | 682 | 82.3 | 17.7 |  |
| Missing | 0 |  |  |  |
| **Late ART initiation** |  |  |  |  |
| No | 2,342 | 83.2 | 16.8 | <0.001 |
| Yes | 1,784 | 97.1 | 2.9 |  |
| Missing | 42 |  |  |  |
| **Total** | 4,168 | 88.8 | 11.2 |  |

Table S2: Patient outcomes following ART initiation using clinic attendance records

| **Events** | **Total** | **Cumulative incidence (95% CI) at 6 months (%)** | **Cumulative incidence (95% CI) at 1 year (%)** | **Cumulative incidence (95% CI) at 2 years (%)** | **Cumulative incidence (95% CI) at 3 years (%)** |
| --- | --- | --- | --- | --- | --- |
| LTFU | 966 | 10.8 (9.8-11.8) | 21.3 (19.9-22.8) | 33.7 (31.8-35.7) | 41.0 (38.5-43.4) |
| Dead | 28 | 0.35 (0.19-0.59) | 0.67 (0.43-1.02) | 0.75 (0.47-1.13) | 1.88 (1.03-3.17) |
| Documented transfer | 4 | 0.09 (0.03-0.25) | 0.13 (0.04-0.86) | 0.13 (0.04-0.86) | 0.13 (0.04-0.86) |

Table S3. Patient outcomes after LTFU ascertained through record linkage with the AHDSS

| **Events** | **Total** | **Cumulative incidence (95% CI) at 6 months (%)** | **Cumulative incidence (95% CI) at 1 year (%)** | **Cumulative incidence (95% CI) at 2 years (%)** | **Cumulative incidence (95% CI) at 3 years (%)** |
| --- | --- | --- | --- | --- | --- |
| Re-engaged | 267 | 16.9 (14.6-19.4) | 23.0 (20.3-25.8) | 32.1 (28.7-35.6) | 38.1 (33.1-43.0) |
| Out-migrated | 298 | 16.9 (14.6-19.4) | 22.8 (20.1-25.5) | 36.4 (32.6-40.1) | 49.4 (43.1-55.3) |
| Dead | 44 | 4.3 (3.1-5.7) | 4.5 (3.3-6.0) | 4.7 (3.5-6.2) | 4.7 (3.5-6.2) |

Table S4. Patient outcomes following ART initiation proportional hazard assumption test

|  | **LTFU** |
| --- | --- |
| Sex & pregnancy status | p=0.001 |
| National origin | p=0.006 |
| Age at ART initiation | p<0.001 |
| Year of ART initiation | p=0.001 |
| Late ART initiation | p=0.394 |

Table S5. Patient outcomes after LTFU proportional hazard assumption test

|  | **Re-engagement** | **Mortality** | **Out-migration** |
| --- | --- | --- | --- |
| Sex & pregnancy status | p=0.566 | p=0.788 | p=0.426 |
| National origin | p=0.068 | p=0.508 | p=0.408 |
| Age at ART initiation | p=0.540 | p=0.002 | p=0.646 |
| Year of ART initiation | p=0.014 | p=0.362 | p=0.473 |
| Late ART initiation | p=0.163 | p=0.400 | p=0.647 |

Table S6. Interaction between age and analysis time on mortality

|  | **Mortality** |  |
| --- | --- | --- |
|  | **Crude HR**  **(95% CI)** | **Adjusted HR**  **(95% CI)** |
| **Age at ART initiation** |  |  |
| <20 years | 0.01 (0.00 - 0.29) | 0.01 (0.00 - 0.45) |
| *Time-varying* | *68.42 (0.82 - 5,732.52)* | *74.92 (0.91 - 6,159.47)* |
| 20-29 | 0.07 (0.02 - 0.31) | 0.11 (0.02 - 0.50) |
| *Time-varying* | *0.15 (0.00 - 168.62)* | *0.17 (0.00 - 193.44)* |
| 30-39 | 0.34 (0.13 - 0.91) | 0.36 (0.13 - 0.99) |
| *Time-varying* | *0.19 (0.00 - 13.10)* | *0.20 (0.00 - 14.32)* |
| 40-49 | 0.41 (0.14 - 1.20) | 0.42 (0.14 - 1.22) |
| *Time-varying* | *0.24 (0.00 - 22.97)* | *0.24 (0.00 - 22.38)* |
| ≥50 years | 1 | 1 |

Figure S1. Predicted hazards of LTFU and 95% CI

Figure S2. Predicted hazards of re-engagement and 95% CI

Figure S3. Predicted hazards of mortality and 95% CI

Figure S4. Predicted hazards of out-migration and 95% CI
